# Editorial for the Special Issue “Antibiotic Prescribing and Antimicrobial Resistance Patterns in Pediatric Patients”

**DOI:** 10.3390/antibiotics12091390

**Published:** 2023-08-31

**Authors:** Costanza Vicentini, Carla Maria Zotti

**Affiliations:** Department of Public Health and Paediatrics, University of Turin, Via Santena 5 bis, 10126 Turin, Italy; carla.zotti@unito.it

Antibiotic overuse is among the most important factors contributing to the growing problem of antimicrobial resistance (AMR). Improving prescribing practices and promoting a more judicious use of antibiotics are both recognized as patient safety and public health priorities. In Italy, almost half of all patients admitted to acute-care hospitals receive an antimicrobial [1]. Italy is the fifth-highest antibiotic consumer in Europe, and several AMR pathogens are now considered to be hyper-endemic, contributing to the severe burden of healthcare-associated infections in the county [2,3].

Children are high consumers of antimicrobial agents. Antibiotics are the most commonly prescribed therapeutic agents in the pediatric population worldwide, particularly in Italy, even though 50% of all pediatric antimicrobial prescriptions are estimated to be unnecessary [4,5]. To develop an effective strategy for managing antibiotic resistance, reliable measurements of childhood antimicrobial consumption and prescribing patterns, both in acute settings and in the community, as well as their impact on resistance, are crucial. However, most metrics and indicators applicable to adult patients are not directly applicable to childhood antibiotic use, due to the absence of age-specific, pediatric-defined daily dosages, age-related differences in the prevalence of infections, and differences between adults and children in the prescription patterns [6,7].

This Special Issue aims to provide further insights into childhood prescribing and resistance patterns in both hospital and outpatient settings, and to investigate their impacts on patient care and antibiotic prescribing policies. The Special Issue includes three systematic reviews of the literature, two retrospective cohort studies, and a cross-sectional survey.

Two studies addressed the acute care setting. The first systematic review, by Chiusaroli and colleagues, provides a thorough investigation of the therapeutic options and outcomes for the treatment of children with infections caused by methicillin-resistant *Staphylococcus aureus* (MRSA), methicillin-resistant coagulase-negative *Staphylococci* (MR-CoNS), and vancomycin-resistant *Enterococci* (VRE) [8]. As stated by the authors, the topic is highly relevant, as infections caused by these agents are increasing; further, treatment options for pediatric patients are limited, and recommendations are based on data from studies involving adult patients.

In their retrospective cohort study on the 2019–2020 *Mycoplasma* epidemic in South Korea, Bae et al. investigated treatment options for pneumonia caused by macrolide-resistant *Mycoplasma pneumoniae* (MRMP), which was unresponsive to initial macrolide therapy [9]. A total of 158 patients admitted to a tertiary referral University hospital with refractory pneumonia were included in the study. According to results of this study, a non-macrolide plus steroid regimen achieved the highest treatment success rate.

The second retrospective cohort study aimed to evaluate trends in isolated pathogens and resistance rates among pediatric patients with periorbital cellulitis over twenty years in Taiwan, with the objective of establishing antibiotics for empirical use [10]. Overall, more than one hundred cases were recorded. *S. aureus* was the most commonly isolated pathogen. The authors also found a decreasing efficacy of oxacillin, correlating with an increasing proportion of MRSA infections.

The primary-care setting was addressed in a systematic review summarizing the health economic evidence on antimicrobial stewardship strategies, including point-of-care testing for upper-respiratory-tract infections (URTIs) in pediatric patients. URTIs are among the most important drivers of inappropriate antibiotic consumption among the pediatric population in the outpatient setting, as viral agents are most often responsible for these infections. The review found that point-of-care testing could be useful to discriminate viral from bacterial infections; however, their use should be incorporated into broader antimicrobial stewardship strategies [11].

Addressing a related topic, Bert et al. conducted a systematic review on the issue of antibiotic self-medication among children [12]. In their study, the authors discussed the geographical, social, cultural, and economic variables that may influence parents’ behaviors, which are crucial elements to inform educational interventions aiming to improve parents’ understanding of the risks associated with this practice.

Finally, Oakley et al. conducted a cross-sectional survey among school-aged children of Timor-Leste to investigate community carriage of AMR pathogens, including extended-spectrum beta-lactamase (ESBL)-producing *Enterobacterales* [13]. Among the 621 investigated stool samples, the prevalence of ESBL-producing bacteria was over 8%, suggesting high rates of carriage of AMR pathogens in the community, which is an important implication for empiric therapy recommendations.
